# Enhancing UAV Visual Landing Recognition with YOLO’s Object Detection by Onboard Edge Computing

**DOI:** 10.3390/s23218999

**Published:** 2023-11-06

**Authors:** Ming-You Ma, Shang-En Shen, Yi-Cheng Huang

**Affiliations:** Department of Mechanical Engineering, National Chung Hsing University, Taichung 40227, Taiwan; mmy@dragon.nchu.edu.tw (M.-Y.M.); g111061305@mail.nchu.edu.tw (S.-E.S.)

**Keywords:** UAV, YOLO, object detection

## Abstract

A visual camera system combined with the unmanned aerial vehicle (UAV) onboard edge computer should deploy an efficient object detection ability, increase the frame per second rate of the object of interest, and the wide searching ability of the gimbal camera for finding the emergent landing platform and for future reconnaissance area missions. This paper proposes an approach to enhance the visual capabilities of this system by using the You Only Look Once (YOLO)-based object detection (OD) with Tensor RT^TM^ acceleration technique, an automated visual tracking gimbal camera control system, and multithread programing for image transmission to the ground station. With lightweight edge computing (EC), the mean average precision (mAP) was satisfied and we achieved a higher frame per second (FPS) rate via YOLO accelerated with TensorRT for an onboard UAV. The OD compares four YOLO models to recognize objects of interest for landing spots at the home university first. Then, the trained dataset with YOLOv4-tiny was successfully applied to another field with a distance of more than 100 km. The system’s capability to accurately recognize a different landing point in new and unknown environments is demonstrated successfully. The proposed approach substantially reduces the data transmission and processing time to ground stations with automated visual tracking gimbal control, and results in rapid OD and the feasibility of using NVIDIA Jetson^TM^ Xavier NX by deploying YOLOs with more than 35 FPS for the UAV. The enhanced visual landing and future reconnaissance mission capabilities of real-time UAVs were demonstrated.

## 1. Introduction

Unmanned aerial vehicles (UAVs) have undergone rapid development in the consumer market in recent years. Originally used for military purposes, UAVs are now applied in a variety of industries, including photography, agriculture, search and rescue, surveying and mapping, patrol inspection, and express delivery. Owing to technological advancements, this development in UAV technology has provided the consumer market with smaller, more economical, more advanced, and safer UAVs.

The key technology of UAVs is their ability to hover while maintaining a stable position, a process commonly known as “hovering”. Realized through advanced sensors and control systems, this technology enables UAVs to maintain a stable position under varying wind speeds and altitudes, and other changes in the external environment. With hovering technology, UAVs can execute various tasks, including aerial photogrammetry, surveying, and surveillance. In addition, UAVs are equipped with autonomous takeoff and landing technologies, which enable them to safely operate in a range of environments. Autonomous takeoff technology allows UAVs to automatically complete testing and preparation tasks prior to takeoff, thereby increasing their operational safety and convenience. Furthermore, the autonomous landing technology of UAVs employs high-precision sensors and smart algorithms to detect and identify the surrounding environment, select a suitable landing point, and achieve a stable and precise autonomous landing. Of these two technologies, autonomous landing technology is the more difficult to realize. Because this technology involves the selection of suitable landing points, this study developed the landing point detection technology for UAVs by integrating artificial intelligence recognition technology for landing point detection. Due to any possible environmental disturbance or missing communication signal, the returned UAVs with a better landing place should be assisted by their onboard visual recognition system when the command of return-to-takeoff-place is absent.

In recent years, research in You Only Look Once (YOLO)-based object detection (OD) has seen widespread application in the field of UAVs. In [1], the application of machine-learning-based OD algorithms to locate hovering UAVs was proposed. That study tested the overall efficiency, accuracy, and consistency of five image detection models—namely, SSD MobileNet v1, SSD Inception v2, Faster RCNN Inception v2, YOLOv2, and Tiny YOLO—in UAVs. The experimental results revealed that YOLOv2 exhibited the highest accuracy when flying over a white background, whereas Faster RCNN Inception v2 demonstrated the most satisfactory results in three indicators and two different backgrounds for detecting a target UAV. In [2], YOLOv4 was employed to recognize four types of drones—namely, multirotor, fixed-wing, helicopters, and VTOLs—and to distinguish drones from birds. By changing the number of convolutional layers in the YOLOv4 model, that study obtained more precise and detailed semantic features. Subsequently, the mean average precision, confusion matrix, intersection over union, precision, accuracy, F1-score, and recall were all used to evaluate the modified YOLOv4 network. Relative to the YOLOv4 basic model, the modified YOLOv4 model exhibited a slight overall improvement of 4% in these evaluation criteria. In [3], the performances of multiple versions of the YOLO algorithm for detecting UAVs were used, and distance-wise analysis (i.e., for close, mid, and far ranges) was performed to measure distance-wise image recognition accuracies. The YOLOv5 model successfully differentiated birds from drones in close and mid ranges. By contrast, the newer YOLOv7 model demonstrated a lower drone detection accuracy than the YOLOv5 model in harsh backgrounds, inferring that the YOLOv5 model exhibited superior drone detection performance compared to the YOLOv7 model. In [4], the GGT-YOLO algorithm was proposed as a novel OD technology for drone-based maritime cruising. That study introduced transformers to enhance the feature extraction capability of the model and to improve its detection of small or occluded objects. Additionally, GhostNet was employed to replace the ordinary convolution in the backbone network with linear transformations, thereby reducing the required parameters and computation cost. Under the combined enhancements of the two technologies, the performance of the GGT-YOLO algorithm for drone-based maritime cruising was improved. In [5], a dataset of visual images taken from a UAV with RGB imaging and thermal infrared information was used for detection. That study employed YOLOv5 as the basic network model and a new model with pre-trained model transfer learning from the MS COCO dataset to improve YOLOv5 for human–object detection in an RGBT image dataset. In [6], co-operative navigation was applied to unmanned swarms. After the co-ordinates of the targets were obtained from the YOLO algorithm, visual vectors were constructed by connecting the targets in the covisibility graph of the nodes in the swarm. That study conducted a field test by using two-wheeled robots and one UAV, and the results revealed that the relative attitude error between the nodes was less than 4°. After filtering, the attitude divergence of the low-precision IMU device could be effectively reduced to increase the precision of the attitude estimation in an unmanned co-operative navigation swarm. In summary, the literature indicates that the integration of YOLO-based OD technology in UAVs is presently the trend in research.

Under consideration of the future development and applications of UAVs, a high density of UAV flights may result in airspace traffic congestion. Given that UAVs currently have limited battery life during flight, the development of technology to enable UAVs to autonomously select suitable landing sites in any given environment is essential. Therefore, to prevent damage to the UAV and to the public caused by crashes during UAV flight, the purposes of this study were as follows: (1) to develop image recognition and automated gimbal tracking control technologies in order to support the UAVs’ hovering in unfamiliar environments, and (2) using a multithread programming technique to incorporate OD technology and an allowable time slot for computation to search and determine the nearest and most suitable landing points. In [7,8], image recognition technologies were incorporated into UAVs for landing, and heliports were used as targets for landing point recognition. Accordingly, the present study employed the YOLO model to recognize open areas and heliports. Because the recognition of open areas must involve the simultaneous detection of whether crowds are present within the vicinity of the landing point, the open space recognition characteristics used in this study differed from those used for general image recognition. To facilitate the emergency landing process of UAVs, the open space recognition characteristics must simultaneously serve as a basis for evaluating whether the open area is suitable for landing, detecting multiple landing points, and selecting the optimal landing point. Alternatively, gimbal control can be applied to search for landing points in order to reduce the power consumption caused by UAVs turning and enable the scanning of landing points when the battery is low.

The remainder of this paper is organized as follows: Section 2 describes the fundamentals of the image recognition technology by using YOLO deep-learning networks. Section 3 presents the experimental results. Finally, Section 4 concludes this paper.

## 2. Fundamentals of YOLO Deep-Learning Networks

The development in computer vision technology can be classified into conventional image processing techniques and deep-learning technology. Prior to the invention of graphic processing units, the classification, feature extraction, and region segmentation of images relied on conventional image-processing techniques, which employed inferences based on mathematical theories to accomplish the objective [9]. In convolution-based image processing, the image is fuzzified, and edge detection is conducted to extract features from the image; the extracted features are subsequently employed to facilitate the identification of information in images by the computer. In [10], a noise-filtering method was proposed for quality control in an automated production line. In [11], meter reading was achieved after the position of the pointer was detected using the Hough transform. However, the difficulty of conventional image processing lies in the need to determine which features are crucial in each image. As the number of classification categories increases, the definition of features becomes more complex, and, thus, engineers are required to fine-tune the parameters. 

Deep learning refers to a data-driven neural network model in which a labeled image dataset is used for machine training. Given that deep neural networks are generally composed of multiple convolutional layers, many low-level features can be merged into high-level features. This merging process enables the machine to automatically compute the most descriptive and significant feature for any given classification [12]. The YOLO system combines convolutional layers, pooling layers, and fully connected layers to form a backbone to achieve real-time OD [13]. In [14], the updated version, YOLOv2, was proposed using batch normalization in the backbone with reference to the architectural design of VGG19. In [15], YOLOv3, using Darknet-53, was proposed. YOLOv3 contains a ResNet structure as the backbone. In [16], YOLOv4 was proposed; its architecture comprised a CSPDarknet-53 backbone [17], SPP additional module, and PANet path-aggregation neck. Compared with the previous versions of the YOLO system, YOLOv4 demonstrated considerably higher object recognition and positional accuracy for objects of varying sizes.

**Figure 1:**
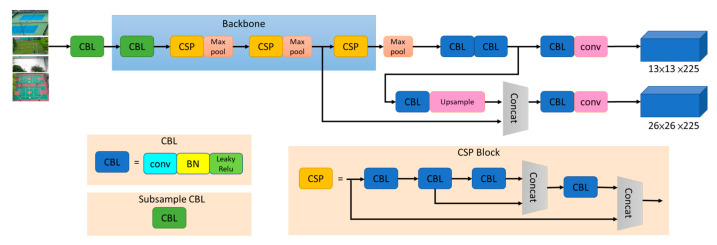
Architecture of YOLOv4-tiny.

**Figure 2:**
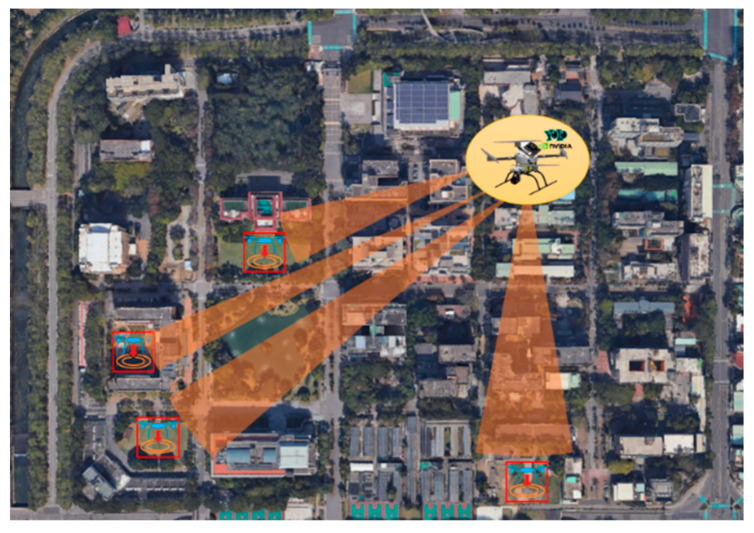
Illustration of landing point detection by the UAV.

Proposed in [18] as a simplified version of YOLOv4, YOLOv4-tiny is a lightweight network architecture designed for low-end GPU devices. As depicted in Figure 1, the architecture comprises seven CBL modules, three CSP modules, three largest pooling layers, an upsample layer, two convolutional layers, and two detection layers of two different dimensions. In YOLOv4-tiny, feature extractions are combined through concatenation instead of adding to simplify the computation and to remove two of the CSP modules from the original YOLOv4 architecture, thereby greatly reducing the computational load. This process enables YOLOv4-tiny to achieve faster execution and training. In the experiment of this study, the UAV is controlled to fly above a school campus. While in flight, gimbal control and YOLO image recognition are used for detecting suitable landing points on campus, as presented in Figure 2.

## 3. Experimentation

Although YOLO has enabled real-time object detection, there are still significant challenges and opportunities for development when applying it to unmanned aerial vehicles. To enhance the intelligence and real-time decision-making capabilities of UAVs during flight, the integration of high-performance GPU and CPU chips into UAVs has emerged as a solution. Consequently, the highly efficient NVIDIA Jeson^TM^ edge-computing devices have garnered attention in the UAV community. NVIDIA is a prominent American technology company specializing in the design of graphics processing units (GPUs) and edge-computing units. The common NVIDIA edge-computing device specifications are shown in Table 1.

In the study of various object detection techniques, Lin demonstrated the feasibility of real-time object detection using the NVIDIA Jetson Nano development kit [19]. In the field of health-monitoring devices, Ahmad similarly employed the NVIDIA Jetson Nano as an experimental platform, due to its stable performance during extended monitoring tasks [20]. Therefore, it is evident that utilizing NVIDIA edge-computing devices for UAVs is indeed feasible. When considering the balance between energy consumption and recognition efficiency in UAVs during hovering with a payload, these edge-computing devices are well-suited for the task.

The NVIDIA Jetson series includes products such as the NVIDIA Jetson Xavier NX, NVIDIA Jetson Nano, and NVIDIA Jetson AGX Orin. Among them, the NVIDIA Jetson Nano is the lightest in weight, with the lowest power requirements and relatively lower computational capability, making it suitable for basic tasks in small UAVs. Although the computational capabilities of the NVIDIA Jetson Xavier NX are lower than those of the NVIDIA Jetson AGX Orin, it weighs only 200 g, making it highly suitable for UAVs for image recognition purposes.

**Table 1:** Comparison of different edge-computing devices [21].

Type	Jetson Xavier NX	Jetson Nano	Jetson AGX Orin
CPU	8-Core ARM Cortex-A57	4-Core ARM Cortex-A57	8-Core ARM Cortex-A78
GPU	NVIDIA Volta	NVIDIA Maxwell	NVIDIA Ampere
Memory	8 GB LPDDR4	4 GB LPDDR4	32 GB LPDDR5
AI performance	21 TOPS	1 TOPS	200 TOPS
Weight	370 g	100 g	1000 g

**Figure 3:**
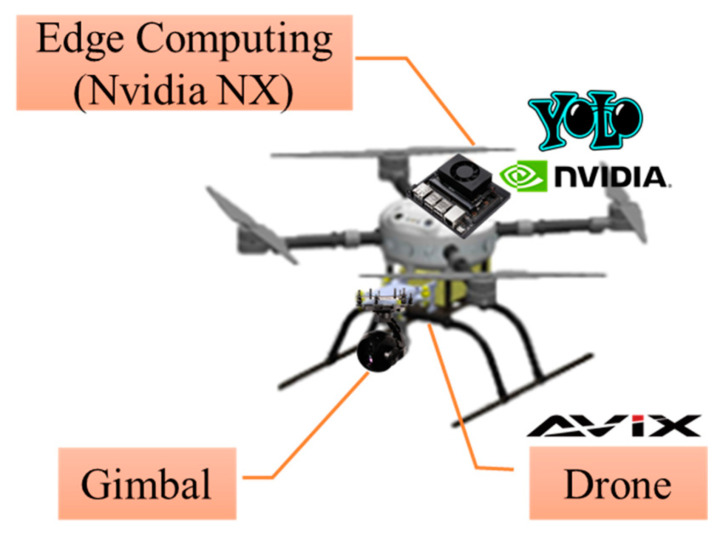
Hardware architecture of the UAV.

**Figure 4:**
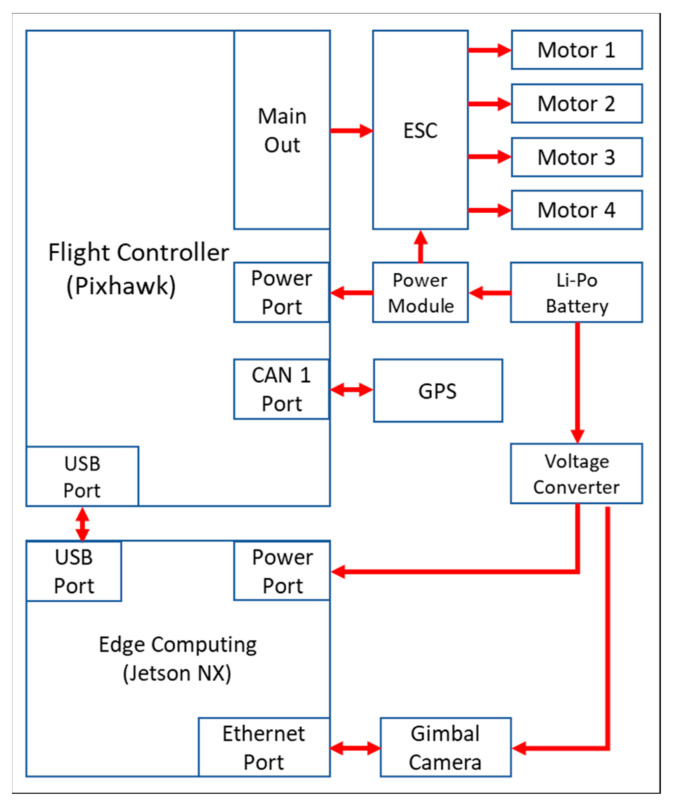
Control signal and communication wiring diagram of the UAV hardware.

The hardware architecture of the experimental UAV in this study comprised the UAV body, a gimbal camera, and an edge-computing module. The UAV used is an AXM-Q7009 4-rotor UAV (AVIX, Taichung, Taiwan) with a diagonal footprint of 702 mm and weight of 2.6 kg. The UAV was powered using a LiPo battery of 17,600 mAH (2 kg) and had a hover time of 30 min, as listed in Table 2. The Pixhawk Cube Orange+ is used as the flight control system. The UAV is equipped with an AXG-AS03F, a mini 3-axis gimbal camera weighing 285 g with dimensions of 96 × 79 × 120 mm. Owing to its lightweight design, the camera is suitable for installation on UAVs with a weight constraint. The camera has 12.71 megapixels and an optical zoom of 3.5 X for image capturing, as detailed in Table 3. In addition, the UAV is equipped with a lightweight, high-efficiency NVIDIA Jetson Xavie NX edge-computing module for YOLO image recognition. Despite having a lower computational capability than the NVIDIA Jetson AGX Orin, the NVIDIA Jetson Xavie NX surpassed its weight by only 200 g, while the NVIDIA Jetson AGX Orin weighs 1 kg. Accordingly, the NVIDIA Jetson Xavie NX is more suitable for installation on UAVs for image recognition. Figure 3 and Figure 4 illustrate the hardware architecture of the UAV and the wiring diagram of the hardware. In Figure 3, the hardware architecture of the NVIDIA Jetson Xavie NX serves as an onboard main control system and is connected to the Pixhawk flight control board through Mavlink for UAV flight. In Figure 4, the NVIDIA Jetson Xavie NX is connected to the gimbal camera through an EtherNet connection and controls the filming direction of the camera. 

**Figure 5:**
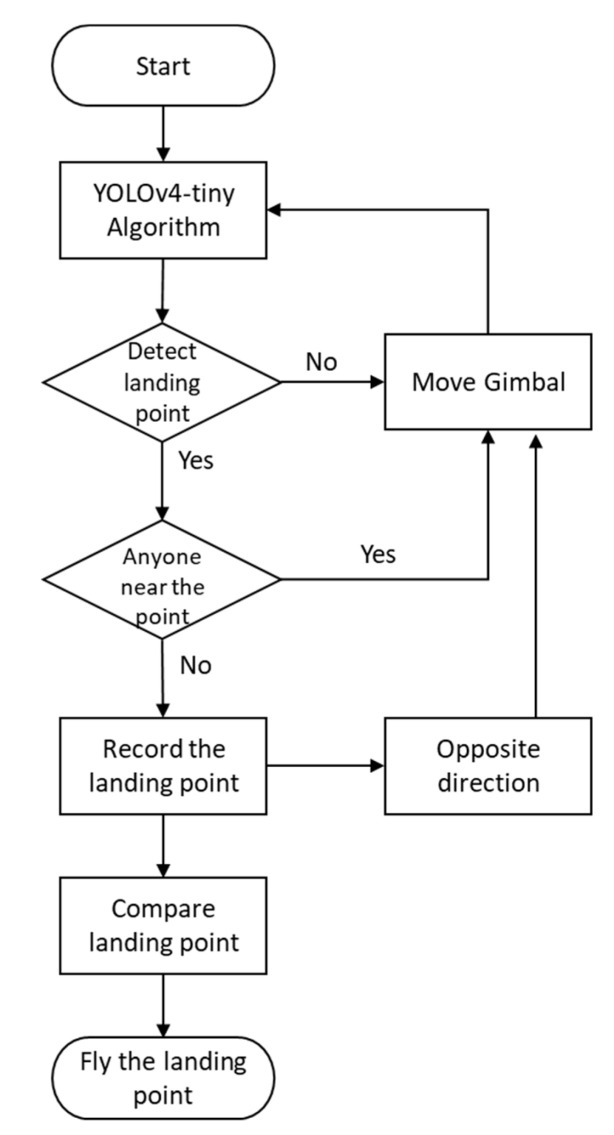
Flowchart of the process of scanning for landing points by the UAV.

As in Figure 5, the flowchart by the tracking gimbal control system for the purpose of searching for a landing point is depicted. The scenarios for the landing point detection process can be achieved as follows: First, the Yolov4-tiny algorithm is activated to search for any possible target landing points. After detecting a landing point, the module can determine whether any humans or other objects are present. If the landing spots are candidates which would not hinder the UAV during landing, the landing point can be recorded as a potential landing point. However, if the algorithm detects humans or other objects that may hinder UAV landing, the automated gimbal tracking control module can adjust the detection angle of the camera and continue the detection process. If all landing spots in the detection direction of the camera is finished, the gimbal control module can turn the gimbal camera toward the opposite direction and continue the detection process. Once the search tracking control is completed, the module can compare all the potential landing points and choose the most suitable one in a future study. 

To enable UAVs to recognize the landing platforms at National Chung Hsing University (NCHU), an edge-computing device (Nvidia Jetson Xavier NX) was equipped on the UAV and a different YOLO architecture was utilized as the object detection solution.

**Figure 6:**
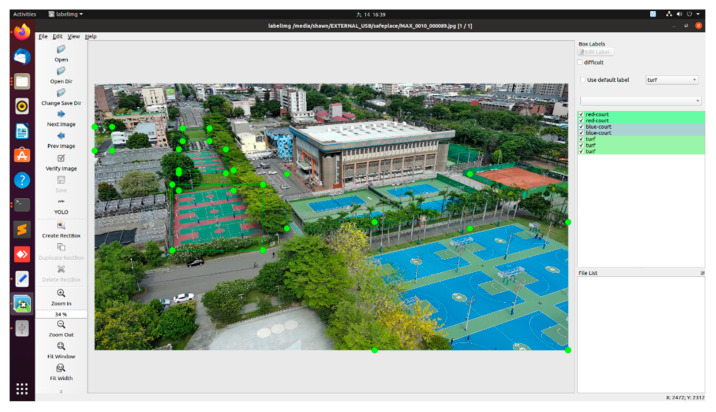
Illustration for labelling NCHU’s turf, red court, and blue court as sports fields.

To identify UAV landing platforms at school, we captured images of lawns, sports fields, skating rinks, playgrounds, and plazas using the UAV as our training dataset for YOLO. The dataset was 707, with 637 images for the training set and 70 images for the validation set. The labeling process is presented in Figure 6.

As mentioned above, the versions YOLOv3 to YOLOv7 are studied in the literature. Based on the same training dataset, the validation of the different YOLOs’ performance in the metrics of precision, recall, and mAP are detailed in Table 4. The presumed results, but with slightly low precision, recall, and mAP, for the test are shown in Table 5. Based on the issues of the training dataset effort, light weight, and power energy consumption for the UAV’s onboard hovering, in this study, the YOLO models, including YOLOv4, YOLOv3, YOLOv4-tiny, and YOLOv3-tiny, are selected and compared by knowing the performance on the edge computer. The training parameters of the neural network were set to be the same, where the batch_size was 16, the subdivisions were 16, the width by height were 416 × 416 pixels, and the iteration was 8000.

In order to improve the FPS performance of the YOLO calculation on the edge computer, the four neural networks were optimized and accelerated using TensorRT, and the recognition time was compared.

## 4. Results

**Figure 7:**
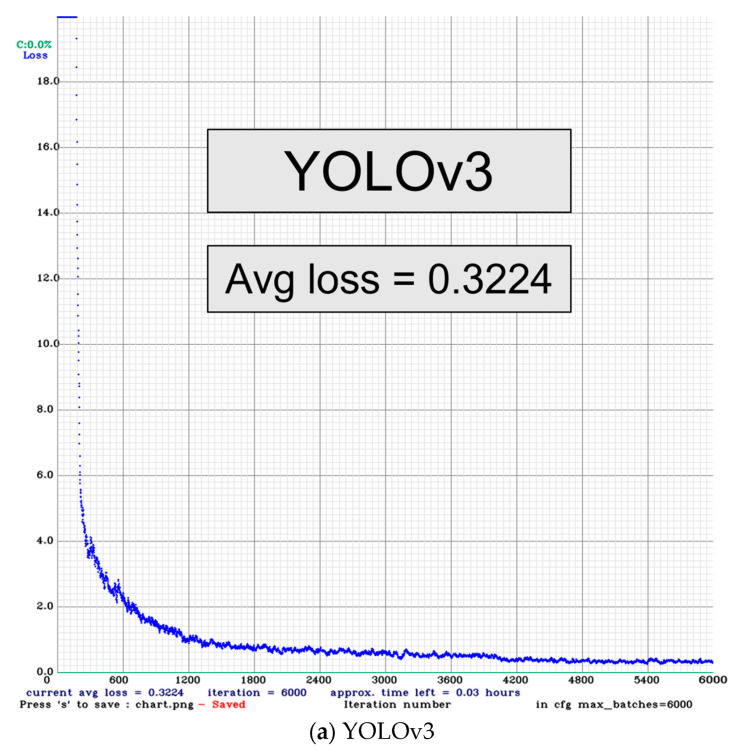

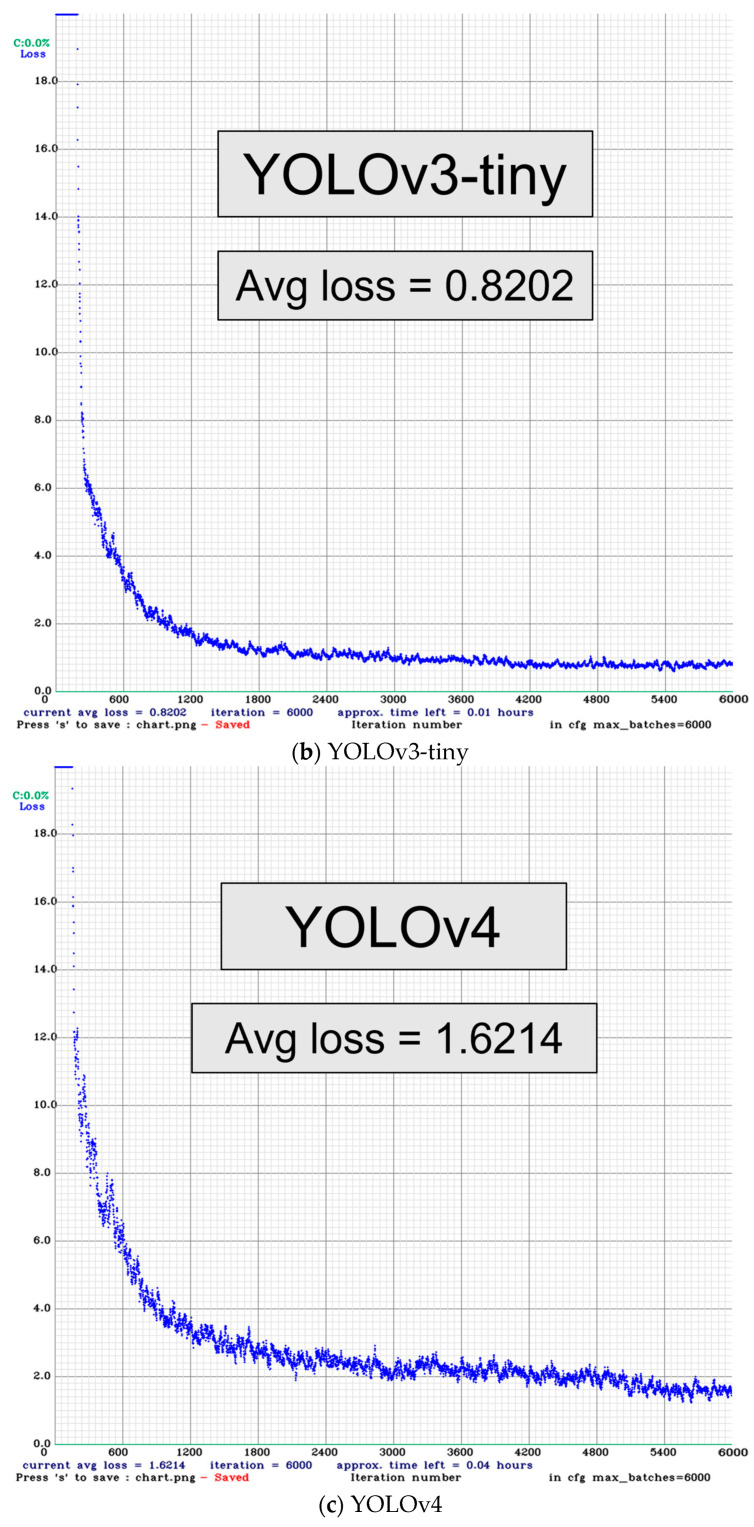

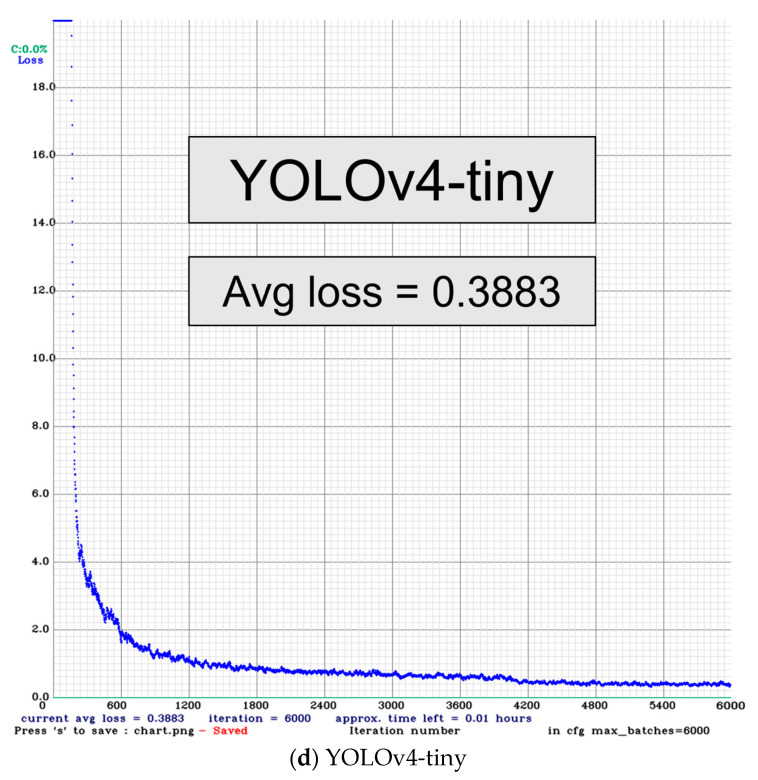
Performance of the loss functions of YOLOv3, YOLOv3-tiny, YOLOv4, and YOLOv4-tiny.

The loss function of the YOLO training was presented in Figure 7. The average precision (AP) and mean average precision (mAP) for the labelled categories are presented in Table 6. From high to low, the accuracy for the YOLOv4, YOLOv3, YOLOv4-tiny, and YOLOv3-tiny are 92.6%, 82.2%, 79.6%, and 63.8%, respectively.

**Figure 8:**
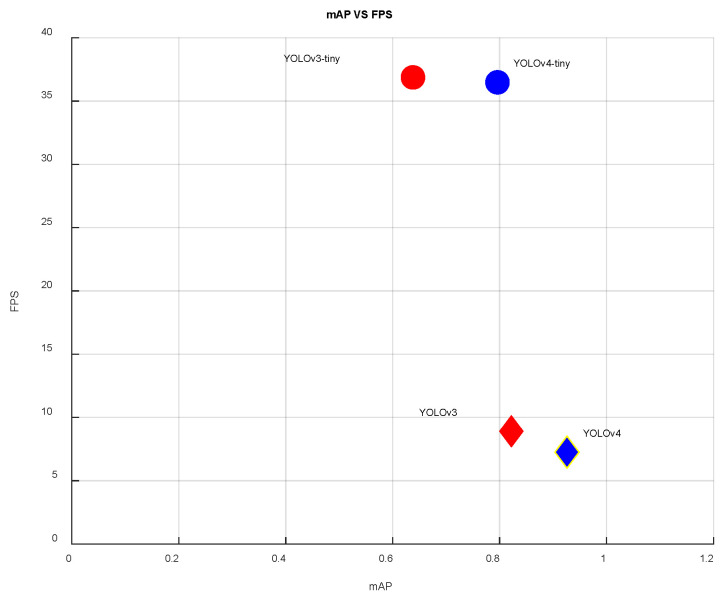
Performance of YOLOv3, YOLOv4, YOLOv3-tiny, and YOLOv4-tiny in mAP and FPS.

**Figure 9:**
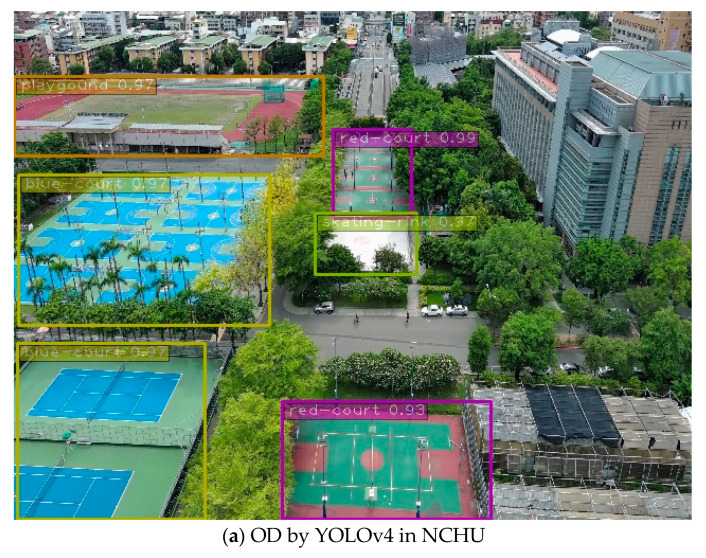

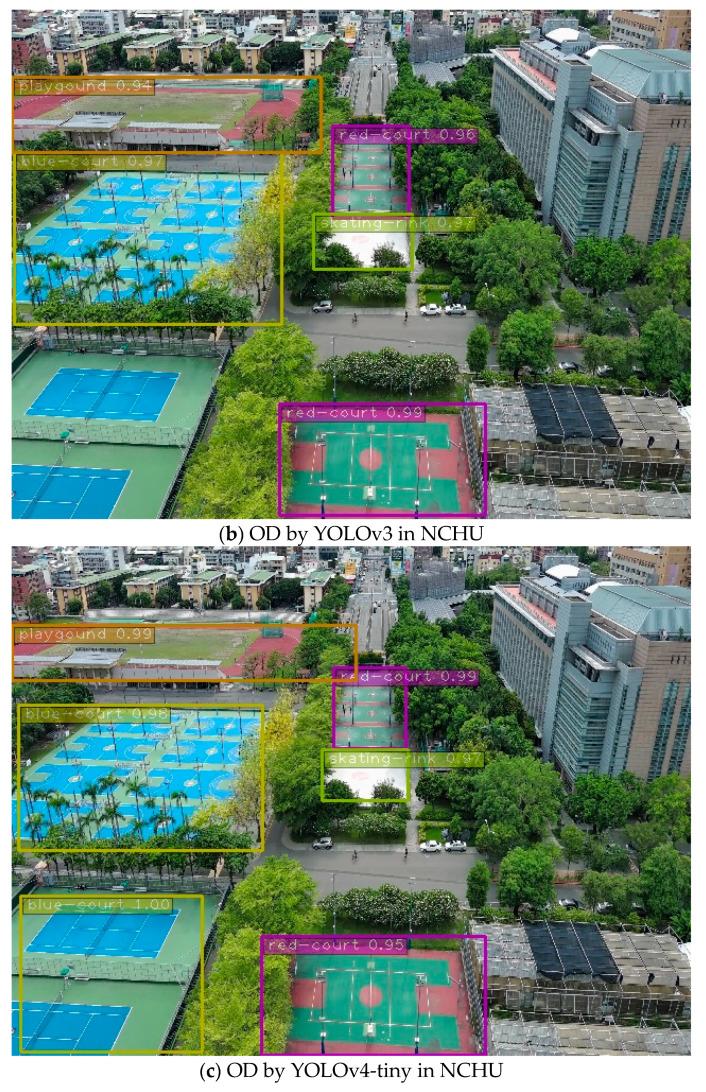

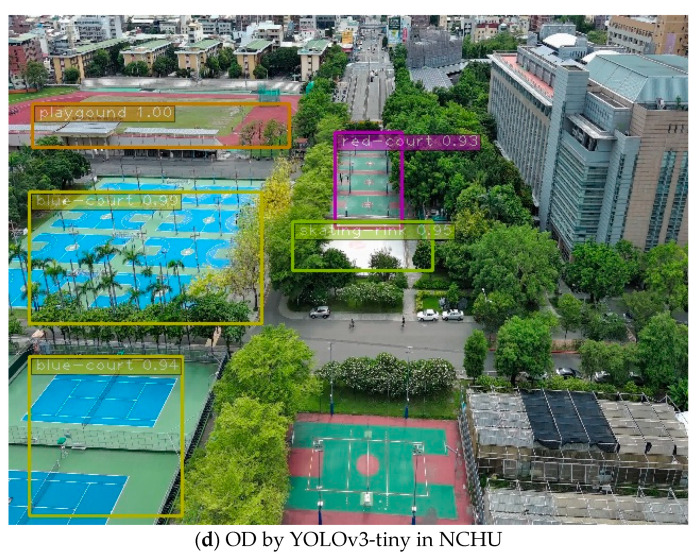
OD images of the actual identification performance in NCHU by (**a**) YOLOv4, (**b**) YOLOv3, (**c**) YOLOv4-tiny, and (**d**) YOLOv3-tiny.

After the YOLO models were accelerated using TensorRT, the mAP for each image recognition on the edge computer was shown in Table 7. As usual, the YOLO-tiny model exhibited a shorter processing time and higher FPS in Table 7. Although YOLOv4-tiny did not achieve the same high accuracy as YOLOv4, it demonstrated the faster recognition and complete labeling of all landing points compared to YOLOv3 and YOLOv3-tiny. In the case of YOLOv4-tiny, the mAP is slightly lower than that of YOLOv3, but the FPS is more than three times higher than YOLOv3. This achievement surpasses the camera’s frame rate limitations while maintaining an almost 80% recognition rate, as presented in Figure 8. The performance of different YOLO models deploying the actual identification process at the National Chung Hsing University (NCHU) is presented in Figure 9. The results for the confidence factor of the actual identification imaging performance at NCHU by (a) YOLOv4, (b) YOLOv3, YOLOv4-tiny, and YOLOv3-tiny structures are more than 93%. 

**Figure 10:**
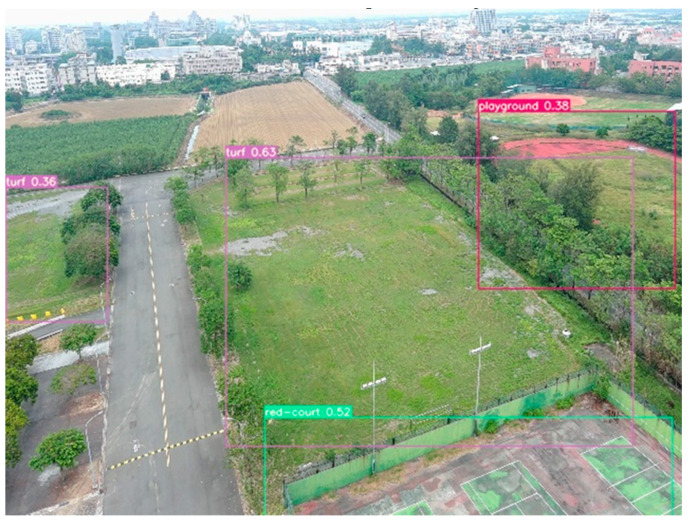

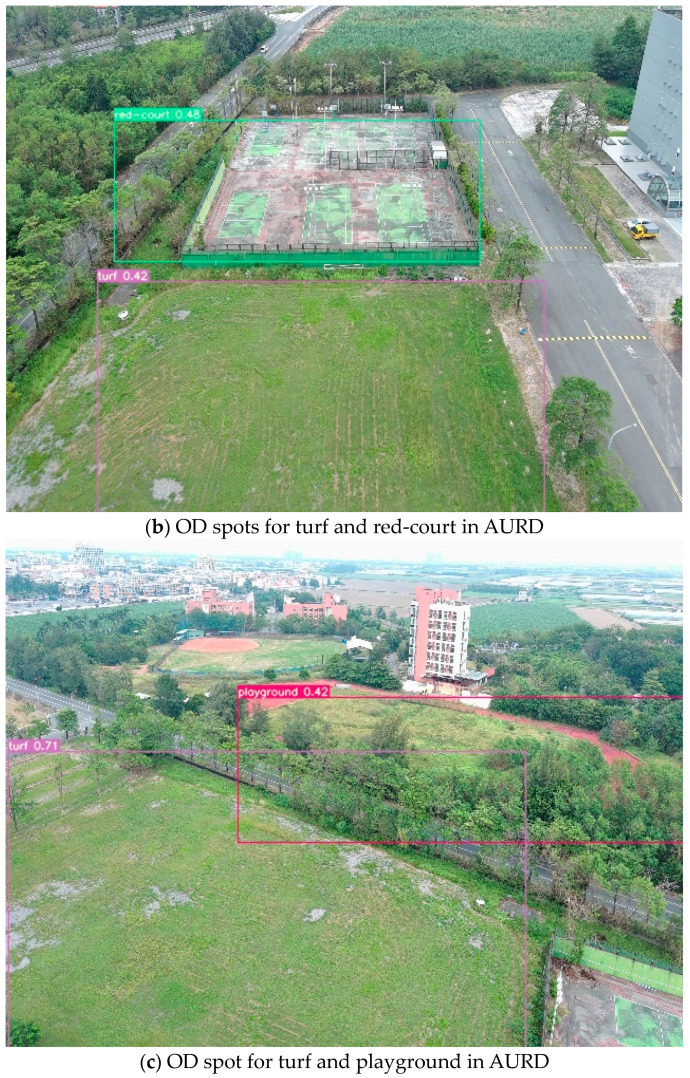
Images of the actual identification performance in AURD with a distance of more than 100 km away from the trained dataset of NCHU.

Illustrating the ability to image the actual identification performance in other fields, the application of the UAV’s OD by developed YOLOs was for the purpose of recognizing the un-pretrained landing point. This was extended to the Asia UAV AI Innovation Application R&D Center (AURD) in Chai-Yi, Taiwan. The distance between the NCHU and the R&D Center is more than 100 km. Although the accuracy may slightly decrease due to the changes in the environment, the experimental data demonstrate that the developed system can effectively label the landing points in various locations. The experimental results show the adaptability to different field conditions by the developed automated tracking gimbal control camera with the YOLO computation (Figure 10).

## 5. Conclusions

This study resolves the solution for the conventional returned UAVs with another new and better landing policy. The developed onboard visual EC system can assist the UAV when the original takeoff point is not available. The gimbal-camera tracking control technology preserves the selection of suitable landing points and we developed a YOLO OD landing point detection technology for UAVs. The computational capacity of the edge computer under the UAV’s payload and battery constraints is resolved by TensorRT. A YOLO-based UAV was deployed with high efficiency and high FPS. In comparison to the general YOLOv4 model, although the YOLOv4-tiny exhibits less accuracy, its FPS performance satisfies the real-time detection requirements, similar to human visual perception. The response time of the onboard EC provides the immediate decision for a ground station when the UAV is on duty. Moreover, the swarm intelligence based on the individual image of the UAV can be synergized. The applied YOLOv4-tiny achieves a 15% higher accuracy, enabling the more effective identification of the landing point when it was compared to the YOLOv3-tiny under the same neural network architecture. 

Therefore, in this study, YOLOv4-tiny with the OD system is suggested to be the most suitable model architecture for object detection for AXG-AS03F UAV. The experimental results also prove that the onboard UAV’s OD can extend to a different location. Overall, this study provides evidence supporting the feasibility and effectiveness of employing YOLO-based object detection and edge computing to enhance the capabilities of the real-time UAV emergency landing operation. 

## Figures and Tables

**Table 2:** Drone specification.

Type	AXM-Q7009
Propellers	4
Diagonal footprint	702 mm
Hover time	30 min
IP rating	IP43
Wind tolerance	Beaufort scale 4
Max. speed	20 m/s
Payload	4 kg
Weight	2.6 kg

**Table 3:** Gimbal specification.

Type	AXG-AS03F
Weight	285 g
Size	96 × 79 × 120 mm
Control range	Tilt: +70°~ −110°, Pan: ±150°
Sensor	CMOS: 1/2.3″, Resoultion: 4024 × 3036
Frame rate	50 Hz/25 fps
Optical zoom	3.5X

**Table 4:** The validation of different YOLOs’ performance in precision, recall, and mAP.

NN Model		Number of Parameters Used in Model	Precision	Recall	mAP@.5	mAP@.5:.95
Large Scale	YOLOv3	61.6 M	0.912	0.928	0.935	0.637
	YOLOv4	64.4 M	0.92	0.936	0.946	0.628
	YOLOv5l	46.5 M	0.926	0.899	0.922	0.612
	YOLOv7	36.9 M	0.91	0.914	0.934	0.631
Small Scale	YOLOv3-tiny	8.6 M	0.894	0.898	0.922	0.585
	YOLOv4-tiny	6.1M	0.899	0.859	0.895	0.518
	YOLOv5n	3.2 M	0.866	0.901	0.927	0.529
	YOLOv5s	12.6 M	0.903	0.924	0.93	0.573
	YOLOv7-tiny	6.2 M	0.905	0.94	0.933	0.614

**Table 5:** The test of different YOLOs’ performance in precision, recall, and mAP in Table 4.

NN Model		Number of Parameters Used in Model	Precision	Recall	mAP@.5	mAP@.5:.95
Large Scale	YOLOv3	61.6M	0.878	0.937	0.923	0.596
	YOLOv4	64.4M	0.87	0.928	0.94	0.609
	YOLOv5l	46.5M	0.876	0.905	0.921	0.598
	YOLOv7	36.9M	0.852	0.941	0.916	0.608
Small Scale	YOLOv3-tiny	8.6M	0.873	0.905	0.898	0.572
	YOLOv4-tiny	6.1M	0.878	0.827	0.859	0.508
	YOLOv5n	3.2M	0.915	0.817	0.88	0.529
	YOLOv5s	12.6M	0.896	0.887	0.938	0.601
	YOLOv7-tiny	6.2M	0.876	0.934	0.91	0.59

**Table 6:** Comparison of different YOLO architectures’ performance for onboard UAV.

Model	Precision	Recall	F1-Score	mAP
YOLOv4	0.9	0.93	0.91	0.926
YOLOv3	0.91	0.77	0.83	0.822
YOLOv4-tiny	0.49	0.87	0.62	0.796
YOLOv3-tiny	0.64	0.70	0.67	0.638

**Table 7:** Comparison of different YOLO architectures’ performance with TensorRT^TM^ optimization.

Model with Tensor RT	Processing Time(s)	FPS
YOLOv4 + TRT	0.1378	7.26
YOLOv3 + TRT	0.1123	8.91
YOLOv4-tiny + TRT	0.0274	36.48
YOLOv3-tiny + TRT	0.0271	36.87

## Data Availability

Not applicable.
